# A Randomized Controlled Trial Comparing Two Wound Dressings Used in Obstetric and Gynecological Surgeries: Trushield NXT vs. Tegaderm

**DOI:** 10.7759/cureus.49207

**Published:** 2023-11-21

**Authors:** Prema D Cunha, Prathima Gowda

**Affiliations:** 1 Obstetrics and Gynaecology, Father Muller Medical College, Mangaluru, IND

**Keywords:** wound healing, wound dressing, trushield, tegaderm, gynecological surgeries

## Abstract

Background

Surgical site infection (SSI) is considered as a healthcare-associated infection. Wound dressings play an imperative role in altering the risk of SSI. In this study, we compared the efficacy and safety of Trushield NXT Non-adherent dressing (Healthium Medtech Limited, Bangalore, India) to the traditional Tegaderm HP+ Pad Film Dressing with Non-Adherent Pad (3M, Bangalore, India) in post-operative wound management of obstetrics and gynecological surgeries.

Methodology

This was a single-centre, prospective, two-arm, parallel-group, randomized, single-blind study conducted between January 2022 and May 2022. One hundred two subjects were enrolled and randomized to receive Trushield or Tegaderm dressing (n=51 each). The incidence of SSI, adverse events (AEs), comfort and performance of the dressings, subject satisfaction, and pain score were assessed in both groups.

Results

The baseline demographics and clinical and physical data were comparable among the groups. During the follow-up study, no SSI or AEs were reported. On the product usage assessment scale, surgeons rated both dressings "good" to "excellent" and favored Trushield for comfortable usage and removal, while Tegaderm was favored for ease of application and flexibility. On the wound pain assessment scale, the proportion of patients reporting "no pain" after surgery was 5.9% in the Trushield group and 4.2% in the Tegaderm group, which increased to 87.8% and 88.6%, respectively, at the end of the follow-up. The subject satisfaction for the comfort and wound healing properties was similar for both products.

Conclusion

Both dressings were equally safe, efficient, and beneficial in the post-operative wound management of obstetric and gynecological surgeries.

## Introduction

Wound healing is a complex phenomenon that involves crosstalk between immunological cells and coagulation factors to regenerate the damaged tissue. A cascade of five synchronized and overlapping events, viz., hemostasis, inflammation, migration, proliferation, and remodeling, occurs during a wound healing process [1]. For effective wound healing, selecting an appropriate wound dressing is important. The dressing protects against trauma, provides physical support, protects against microbial invasion, and absorbs exudate [2,3]. Currently, several types of wound dressing materials are available in the market. In gynecological and obstetrical surgery, empirical hospital practice requires covering the operative site with a proper skin dressing. Surgical wounds or incisions can be healed only after bringing the wound edges together using sutures, staples, or clips, followed by covering with a dressing [4, 5]. The dressing material is selected based on the wound's type, depth, location, and extent. An ideal wound dressing should maintain a moist environment, enable gaseous exchange, promote angiogenesis, manage exudate, reduce pain, and be comfortable during usage and removal [2].

The main problem associated with an invasive surgical procedure is an infection, commonly referred to as surgical site infection (SSI). SSIs account for 3-14% of healthcare-associated infections in subjects who undergo surgical procedures [6, 7]

In modern wound management settings, 3M Tegaderm is an old, trustworthy dressing regularly being used in surgical directorates [8]. It comprises a silicone-coated paper liner, polyurethane film, and a non-adherent pad with a non-woven backing with acrylate adhesive [9]. In 2014, Kerr & and Arrowsmith reported that Tegaderm superabsorber dressing ensures effective clinical outcomes with good exudate management [10]. In another study, Michelin et al. (2020) reported that Tegaderm dressings are most effective in preventing water penetration (waterproof), most comfortable, and cost-effective [11]. Overall, the Tegaderm dressing has the properties of exudate management, protection against infections, breathable for gaseous exchange, waterproof, and comfort. However, experimental and clinical data on the usage of Tegaderm HP+Pad Film Dressing with Non-Adherent Pad are limited to obstetrics and gynecological surgeries.

Recently, Trushield NXT Non-adherent wound dressing with unique "Dimethyl tetradecyl [3-(trimethoxy silyl) propyl] ammonium chloride" (DTAC) technology has emerged (Healthium Medtech Limited, Bangalore). It is an active advanced wound care product that consists of a 3-D knitted hydrocellular textile substrate made of Polyethylene Terephthalate (PET) and Polyurethane (PU). The 3D substrate is permanently bound and cross polymerized-cross linked with DTAC that is immobilized on the substrate and does not leach out of the dressing. This patented anti-microbicidal technology provides continuous infection protection, allows exudate and moisture management, is non-adherent to the wound, and is waterproof (United States Patent No. US9,566, 363 B2, 2017) [12].

In this regard, a randomized controlled study was conducted to compare the efficacy and safety of two non-adherent surgical wound dressings, i.e., Trushield NXT dressing (Healthium Medtech Limited, Bangalore, India) with Tegaderm HP+Pad Film Dressing with Non-Adherent Pad (3M, Bangalore, India) in the post-operative wound management of obstetrics and gynecological surgeries.

## Materials and methods

Trial design

This was a single-center, prospective, two-arm, parallel-group, randomized (1:1), single-blind study to compare the efficacy and safety of two non-adherent dressings, i.e., Trushield NXT Non-adherent dressing and Tegaderm HP+Pad Film Dressing with Non-Adherent Pad. The study was conducted over 14 weeks, from January 2022 to May 2022.

Ethics

The study protocol was approved by the Institutional Ethics Committee of Father Muller Medical College, Mangalore approved the study with registration number FMIEC/CCM/2125/2021 dated 16th Dec 2021, and it was submitted and registered prospectively with the Clinical Trial Registry of India before initiation of the trial [CTRI/2021/12/038884 (Registered on: 23/12/2021)]. It was also registered in the Research Registry (unique identifying number (UIN): researchregistry8763) [13]. The study was conducted in compliance with Good Clinical Practice guidelines. A written informed consent was obtained from each participant prior to their enrolment. The study was reported per the 2010 Consolidated Standards of Reporting Trials (CONSORT) guideline for clinical trials [14].

Participants

One hundred two subjects were enrolled in the Department of Obstetrics and Gynecology, Father Muller Medical College, Mangalore. Female subjects aged 18-65 years who underwent uncomplicated open obstetric/gynecological surgery and had hemoglobin (Hb) levels of > 9gm/dl were included in the study. Conversely, subjects with eczema, peripheral vascular disease, immunosuppressive or corticosteroids, systemic infection, active neoplastic condition, and uncontrolled diabetes were excluded from the study. 

Interventions

Subjects were randomized to receive either Trushield NXT Non-adherent dressing or Tegaderm HP+Pad Film Dressing with Non-Adherent Pad for the post-operative wound management of obstetrics and gynecological surgeries.

Data collection and outcome measures

Demographic data, including age, weight, height, ethnicity, obstetric history, medical history, and indication for surgery, was obtained at baseline. Further, the physical and clinical examinations were conducted at baseline and in all in-person post-surgery follow-ups (Day 3±1 & 35±7). Surgical details such as type of surgery, length of incision, suture used for closing the skin, type of suturing, and the number of blood transfusions required were recorded on the day of the surgery.

Objectives

Primary Objective

The study's primary objective was to assess the effectiveness of Trushield NXT Non-adherent wound dressing versus Tegaderm HP+Pad Film Dressing with Non-Adherent Pad in post-operative wound management of obstetric and gynecological surgeries. The effectiveness was evaluated by assessing the incidence of SSI using the Centers for Disease Control and Prevention (CDC) criteria.

Primary Endpoint

The incidence of SSI was assessed using the CDC criteria among the two groups during all the post-surgery follow-ups (Day 3±1, 10±1 & 35±7). The CDC criteria classify Superficial incisional SSI as an infection involving skin and subcutaneous tissue of the incision that occurs within 30 days after any NHSN operative procedure [15].

Secondary Objectives

The secondary objectives include the assessment of post-operative wound complications, scar cosmesis, subject and surgeon satisfaction with the dressing, wound pain, and pain during dressing removal.

Secondary Endpoints

Data including pain score, product usage assessment, subject satisfaction on the dressing and wound healing, and Modified Stony Brook Scar Evaluation were assessed and compared for measuring the efficacy of Trushield NXT Non-adherent wound dressing vs. Tegaderm HP+Pad Film Dressing with Non-Adherent Pad. The condition of the surrounding skin, wound separation, and adverse events (AEs) were also noted.

Product Usage Assessment

Surgeons rated the product during application (ease of application, flexibility of the dressing), usage (exudate management, breathability of skin, conformance to skin, stickiness of adhesive layer, waterproofing property), and during removal (ease of removal and non-adherence to wound) on a scale of 1 (poor) to 5 (excellent). The ratings for the application of the dressing were given on the day of surgery. Further ratings on the usage and removal of the dressing were given on post-surgery follow-up (Day 3±1).

Wound Pain Score

The subjects reported wound pain on a scale of 0 (no pain) to 10 (worst pain) using the 0-10 numeric pain rating on a visual analog scale (VAS) on the day of surgery and all the post-surgery follow-ups (Day 3±1, 10±1 & 35±7). The number of analgesics prescribed was also documented, along with the wound pain score.

Subject Satisfaction

The subjects were asked to rate the comfort of dressing in terms of usage and removal on a scale of 1 (poor) to 5 (excellent). Their ratings were obtained during post-surgery follow-up (Day 3±1). Further, ratings on their satisfaction with wound healing on a scale of 1 (poor) to 5 (excellent) were also obtained during post-surgery follow-ups (Day 10±1 & 35±7).

Modified Stony Brook Scar Category

The scar category was evaluated during the last follow-up (Day 35±7) and compared using the modified Stony Brook Scar Evaluation Scale (SBSES) among the two groups [16] on a scale of 0 (prominent) to 2 (absent). The modified SBSES includes width (0, scar widening prominent and >2 mm; 1, scar widening present and ≤ 2 mm; 2, no scar widening), height (0, prominent scar elevation; 1, scar elevation present; 2, no scar elevation), color (redness) (0, scar prominently redder than the surrounding; 1, scar redder than the surrounding; 2, scar of the same color as or lighter than the surrounding skin), and visibility of the incision line (0, prominent incision line; 1, incision line present; 2, incision line absent).

Sample size estimation

The sample size was calculated using Laura Flight and Steven A. Julious's Practical guidelines [17]. It was estimated that 92 subjects (46 in each group) would be sufficient to detect a difference of 10% between groups in the proportion of subjects with better wound healing, with 80% power and 5% significance level. Considering the 10% dropout, the total number of subjects included in the study was 102 (51 in each group).

Randomization and dressing allocation

After recruitment of the study subjects, they were randomized using the block randomization technique in a ratio of 1:1 to ensure unbiased and balanced allocation of participants to each dressing arm, i.e., Trushield NXT Non-adherent wound dressing or Tegaderm HP+Pad Film Dressing with Non-Adherent Pad. An independent statistician generated a computer-based, automated randomization number. Before surgery, the randomization codes were issued sequentially numbered, opaque sealed envelopes (SNOSE) to allocate the dressing arm.

Statistical analysis

Categorical data were compared using the chi-square (χ2) t-test, and continuous data were compared using the student's t-test. All statistical analyses were carried out using GraphPad version 5.1. The p-value of <0.05 was considered statistically significant.

## Results

**Figure 1:**
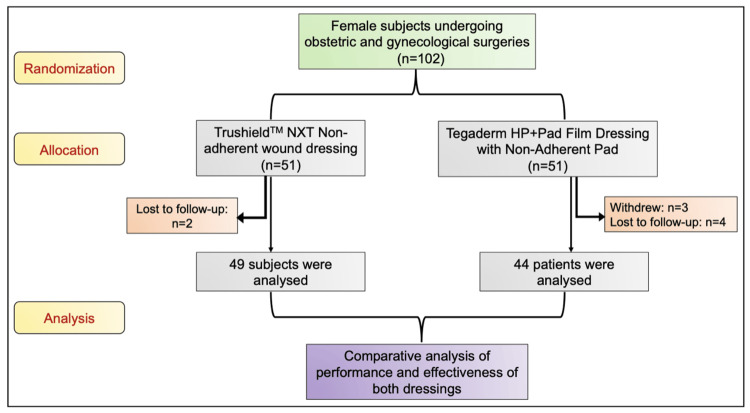
CONSORT Flow diagram

One hundred two female subjects were enrolled and subjected to randomization, with 51 females in each dressing arm. Of 102 subjects, three subjects withdrew from the study, and six subjects were lost to follow-up. Last follow-up data (after 35 days) was not available for the subjects who were lost to follow-up (Figure 1).

The study was conducted from 1st Jan 2022 to 5th Apr 2022 at Father Muller Medical College, Mangalore. The final follow-up was completed on 11th May 2022.

Demographics

The demographic data in both groups were similar with respect to age, weight, height, BMI, obstetric history, and medical history (Table 1).

**Table 1:** Demographic, medical history, and surgical details of the subjects HTN= Hypertension, DM= Diabetes mellitus, LSCS= lower (uterine) segment Caesarean section, kg= kilograms, cm= centimetres, m= metres, n= number of subjects, %= percentage

Parameters (Mean ± SD)	Trushield group (n=51)	Tegaderm group (n=48)	P-value
Age, years	35.1 ± 9.43	34.2 ± 9.91	0.638
Weight, kg	64.2 ± 9.10	63.8 ± 11.34	0.844
Height, cm	154.8 ± 7.04	155.6 ± 6.77	0.588
BMI, kg/m^2^	26.9 ± 3.99	26.3 ± 4.34	0.467
Medical history, n (%):			0.437
HTN	04 (7.8)	06 (12.5)	
DM	03 (5.9)	01 (2.1)	
Hypothyroidism	07 (13.7)	03 (6.3)	
Asthma	01 (2)	03 (6.3)	
Type of surgery, n (%):			0.176
Elective LSCS	27 (52.9)	19 (39.6)	
Emergency LSCS	08 (15.7)	16 (33.3)	
Hysterectomy	13 (25.5)	11 (22.9)	
Laparotomy	03 (5.9)	02 (4.2)	
Length of incision, cm, n (%):			0.763
12	24 (47.1)	22 (45.8)	
13	23 (45.1)	20 (41.7)	
14	04 (7.8)	06 (12.5)	

Moreover, among the groups, no significant difference was observed in the clinical parameters such as temperature, respiratory rate, heart rate, and blood pressure (p>0.05). The general physical and clinical examination of all the recruited subjects was normal and comparable between the groups. 70.7% of subjects underwent lower (uterine) segment Caesarean section (LSCS; elective or emergency), 24.2% of subjects underwent hysterectomy surgeries, and 5.1% of subjects underwent laparotomy. In all the subjects, continuous sub-cuticular suturing was carried out using poliglecaprone-25 sutures. More than 80% of the subjects did not have blood transfusions.

Primary endpoints

Surgical Site Infection

The incidence of SSI using CDC criteria among the two groups was assessed. No incidence of SSI was observed in any of the groups during the follow-up period of 14 weeks.

Secondary endpoints

Wound Pain Assessment

**Figure 2:**
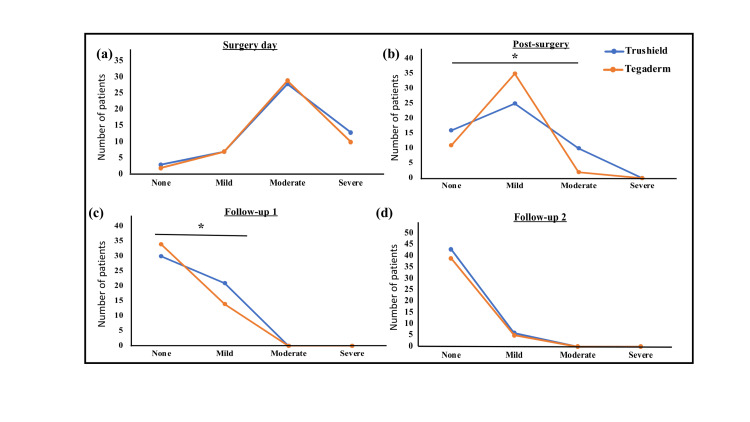
Wound pain assessment among the groups Line graphs represent the proportions of patients in different groups with wound pain categorized as none, mild, moderate, and severe. Wound pain assessment during (a) Surgery day, (b) Post-surgery, (c) Follow-up 1 (Day 10), and (d) Follow-up 2 (Day 35); *p < 0.05.

Post-operative pain on the day of surgery was not significantly different between the two groups (P value: 0.506). Post-surgery on Day 3, the proportions of subjects reporting "no pain" were 31.4% subjects in the Trushield group and 22.9% subjects in the Tegaderm group; "mild pain": 49% and 72.9%, and "moderate pain": 19.6% and 4.2% subjects, respectively. During the Follow-up visit on Day 10, the proportion of subjects reporting "no pain" increased to 58.8% subjects in the Trushield group and 70.8% subjects in the Tegaderm group, while the percentage decreased in "mild pain" category to 41.2% and 29.2%, respectively. During the follow-up visit on Day 35, the proportion of subjects reporting "no pain" was statistically similar between both groups (87.8% vs. 88.6%; P value: 0.169). The number of analgesics used was similar in both groups during the study. The wound pain assessment data is shown in Figure 2.

Product Usage Assessment

On the product usage assessment scale, surgeons rated both dressings between 3 to 5 (good to excellent). When the ratings of both the dressings were compared, Trushield wound dressing was found better than Tegaderm in terms of exudate management (60.8% vs. 52.1%), breathability of skin (29.4% vs. 18.7%), conformance to skin (52.9 vs. 41.6%), waterproofing property (49.1% vs. 43.7%), ease of removal (51% vs. 39.6%), and non-adherence to wound (45.1% vs. 37.5%) (p<0.05). However, Tegaderm was rated better for ease in application (35.4% vs. 29.4%), flexibility of the dressing (37.5% vs. 23.5%), and stickiness of the adhesive layer (58.3% vs. 41.2%) (p<0.05). The product assessment data are summarized in Table 2.

**Table 2:** Surgeon’s responses on product usage assessment n= number of subjects, %= percentage; <0.05: favoured Trushield NXT wound dressing; <0.05*: favoured Tegaderm

Product usage assessment scale (1-5: 1-poor; 5-excellent)	Trushield group (n=51)	Tegaderm group (n=48)	P-value
Surgery day			
Surgeon’s response:			
Ease			<0.05*
Very good	36 (70.6)	31 (64.5)	
Excellent	15 (29.4)	17 (35.4)	
Stretchability/Flexibility			<0.05*
Good	05 (9.8)	04 (8.3)	
Very good	34 (66.7)	26 (54.2)	
Excellent	12 (23.5)	18 (37.5)	
Time taken for application of dressing (sec)	51.7 ± 18.15	51.3 ± 16.59	1.000
Post-surgery			
Surgeon’s response:			
Exudate management			<0.05
Good	01 (2)	04 (8.3)	
Very good	31 (60.8)	25 (52.1)	
Excellent	19 (37.2)	19 (39.6)	
Breathability of skin			<0.05
Good	02 (3.9)	01 (2.1)	
Very good	34 (66.7)	38 (79.2)	
Excellent	15 (29.4)	09 (18.7)	
Conformance to skin			<0.05
Good	02 (3.9)	05 (10.5)	
Very good	27 (52.9)	20 (41.6)	
Excellent	22 (43.1)	23 (47.9)	
Stickiness of adhesive layer			<0.05*
Good	05 (9.8)	03 (6.3)	
Very good	25 (49)	17 (35.4)	
Excellent	21 (41.2)	28 (58.3)	
Water proofing property			<0.05
Good	04 (7.8)	04 (8.3)	
Very good	22 (43.1)	23 (48)	
Excellent	25 (49.1)	21 (43.7)	
Ease of removal			<0.05
Good	03 (5.9)	04 (8.3)	
Very good	22 (43.1)	25 (52.1)	
Excellent	26 (51)	19 (39.6)	
Non-adherent			<0.05
Good	01 (2)	02 (4.2)	
Very good	26 (51)	28 (58.3)	
Excellent	23 (45.1)	18 (37.5)	

Further, the time taken for dressing application was found to be similar in both groups, i.e., approximately 51 seconds.

Next, subject satisfaction during dressing usage and removal was evaluated and compared among both groups. As per the subject ratings, no significant difference was found between the two products, and they were comparable in usage and removal. The proportion of subjects reporting "no pain" during the removal of the dressing was 31.4% in the Trushield group and 27% in the Tegaderm group. Further, the subject satisfaction with wound healing did not differ among the groups during the follow-up period. The details are given in Table 3.

**Table 3:** Subject satisfaction on product usage and wound healing n= number of subjects, %= percentage

Parameters, n (%)	Trushield group (n=51)	Tegaderm group (n=48)	p-value
Follow-up 1			
Comfortable usage			0.063
Good	-	03 (6.3)	
Very good	29 (56.9)	20 (41.6)	
Excellent	22 (43.1)	25 (52.1)	
Comfortable removal			0.754
Good	02 (3.9)	01 (2.1)	
Very good	28 (54.9)	28 (58.3)	
Excellent	21 (41.2)	19 (39.6)	
Pain during dressing removal			<0.05
No pain	16 (31.4)	13 (27)	
Low	32 (62.7)	34 (70.8)	
Moderate	03 (5.9)	01 (2.1)	
Satisfaction with wound healing (1-5 scale)			0.259
Good	03 (5.9)	04 (8.3)	
Very good	36 (70.6)	32 (66.7)	
Excellent	12 (23.5)	12 (25)	
Follow-up 2			
Satisfaction with wound healing			0.236
Good	02 (3.9)	02 (4.6)	
Very good	29 (56.9)	25 (56.8)	
Excellent	18 (36.7)	17 (38.6)	

Wound Conditions and AEs

The condition of the skin surrounding the incision was assessed and found to be healthy in all the subjects. At the end of the study, no wound separation, redness, tenderness, or swelling in the incision area was observed in any of the subjects belonging to the two groups. Moreover, no AEs were reported by any subject at any time during the study.

Scar Category

The scores of modified SBSES were summarized in Table 4.

**Table 4:** Scar evaluation among the groups n= number of subjects, %= percentage.

Modified Stony Brooks Scar evaluation scale	Trushield group (n=49)	Tegaderm group (n=44)	p-value
Width			0.061
Scar width greater than or equal to 2mm	02 (3.9)	02 (4.6)	
Scar width lesser than or equal to 2mm	10 (19.6)	10 (22.8)	
No scar widening	37 (75.5)	32 (72.6)	
Height			<0.05
Prominent elevation	-	03 (6.8)	
Elevation present	12 (24.5)	11 (25)	
No scar elevation	37 (75.5)	30 (68.2)	
Color (redness)			<0.05
Prominent	-	01 (2.3)	
More red than the surrounding skin	09 (10.3)	09 (20.4)	
Same colour	40 (89.7)	34 (77.3)	
Incision line			0.853
Prominent	-	01 (2.3)	
Present	46 (95.8)	42 (95.4)	
Absent	03 (4.2)	01 (2.3)	

Compared to the Tegaderm group, the Trushield group showed better results for the healed wound with respect to the height of the scar (75.5% vs. 68.2%) and redness of the surrounding skin (89.7% vs. 77.3%).

## Discussion

This is the first clinical study that predominates the clinical use of a new DTAC technology-based wound dressings in obstetric and gynecological surgeries. The results of this study provide substantial evidence on the performance, safety, and effectiveness of Trushield NXT Non-adherent wound dressing.

Primarily, the subjects in both the dressing arms, i.e., Trushield NXT Non-adherent wound dressing vs. Tegaderm HP + Pad Film Dressing with Non-Adherent Pad, had no significant differences in demographics, physical, and clinical parameters (Table 1). Also, the proportion of subjects with medical history did not differ in both groups, indicating that these factors did not correlate to affect the performance of the dressings. Next, the wound pain was assessed after surgery and during the follow-ups. After surgery, the reported wound pain was similar in both the dressing arms on the day of surgery. Post-surgery on Day 3, the proportions of subjects reporting "no pain" were 31.4% subjects in the Trushield group and 22.9% subjects in the Tegaderm group; "mild pain": 49% and 72.9%, and "moderate pain": 19.6% and 4.2% subjects, respectively.

Further, the wound healing capability of Trushield NXT Non-adherent wound dressing and Tegaderm HP + Pad Film Dressing with Non-Adherent Pad was also similar (88.2% of subjects in Trushield group vs. 85.4% of subjects in Tegaderm group). In a prospective randomized controlled trial, Ravenscroft et al. (2006) compared wound dressings used in hip and knee surgery and found that the wound healing capacity of Tegaderm is approximately 82% [8]. In another randomized controlled trial, Terill et al. (2007) compared wound healing in split-thickness skin graft donor sites and observed that 79% of the donor sites were healed completely with Tegaderm Absorbent polyurethane film dressing [18]. Both these studies are in concordance with our findings. At the end of the study, the proportion of subjects reporting "no pain" was statistically similar in both groups, suggesting comparable wound healing and pain reduction.

Further, surgeons rated Tegaderm better regarding ease of application and flexibility during the dressing application. Trushield dressing was rated better than Tegaderm dressing in terms of exudate management, the breathability of skin, conformance to the skin, waterproofing property, ease of removal, and non-adherence to wound (p<0.05). The time taken for application of the dressing was 51 seconds in both groups.

All the subjects found both the dressings comfortable during usage and removal. There was no significant difference in the comfort experienced by subjects belonging to both groups during the usage and removal of the dressing. In both groups, the subjects were similarly satisfied with the wound healing during the study period. In a prospective randomized trial, Brown-Etris et al. (2008) evaluated and measured subject comfort dressing performance and wound healing in managing pressure ulcers. The authors favored Tegaderm Absorbent acrylic dressing over hydrocolloid dressing in terms of absorption, comfort, ease of removal, and non-adherence to the wound [19]. In a retrospective review of 99 subjects, Fakhoury et al. (2019) showed high subject satisfaction when using Tegaderm for vascular surgery incisions [20]. This finding is in concordance with our results that suggest subjects' overall comfort and satisfaction with Tegaderm dressing, which was similar to Trushield NXT Non-adherent wound dressing.

Scaring was evaluated at the end of the study protocol using the modified Stony Brook Scar Evaluation Scale (SBSES) [16]. The modified SBSES is based on four parameters: width, height, color, and visibility of the incision line. Regarding all height and color parameters, Trushield NXT Non-adherent wound dressing was found to be better than Tegaderm dressing. This study has a few limitations. The sample size was small and demographically based as the study was single-centric.

## Conclusions

With respect to the primary endpoint, using both the wound dressing resulted in no surgical site infections or any adverse events. In the final analysis of secondary endpoints concerning effectiveness, performance, and wound healing properties, Trushield NXT Non-adherent wound dressing was rated better in terms of exudate management, breathability of skin, waterproofing property, ease of removal, non-adherence to wound, and SBSES score while Tegaderm was rated better for ease in application, adhesiveness, and flexibility during the application of the dressing. All the subjects belonging to the groups were equally satisfied with their respective dressings. Conclusively, Trushield NXT Non-adherent wound dressing and Tegaderm HP+Pad Film Dressing with Non-Adherent Pad dressings were found equally suitable, safe, beneficial, and efficient in the post-operative wound management of obstetric and gynecological surgeries.
